# Ultrasonographic Morphology of the Abdominal Organs of the White-Eared Opossum (*Didelphis albiventris*)

**DOI:** 10.3390/ani16152392

**Published:** 2026-08-03

**Authors:** Eloara Giovanna Aguiar Bonassoli de Oliveira Martins, Rodrigo Antonio Martins de Souza, Luciana do Amaral Oliveira, Carla Fredrichsen Moya

**Affiliations:** 1 Postgraduate Program in Veterinary Sciences, State University of the Midwest—Unicentro, Guarapuava 85040-167, Brazil; veteloaragiovanna@gmail.com; 2 Department of Veterinary Medicine, State University of the Midwest—Unicentro, Guarapuava 85040-167, Brazil; rdesouza@unicentro.br (R.A.M.d.S.); lucianaoliveira@unicentro.br (L.d.A.O.)

**Keywords:** abdominal ultrasound, diagnostic imaging, ultrasound anatomy, white-eared opossum

## Abstract

Ultrasound is a fundamental diagnostic imaging tool in veterinary medicine that allows for the noninvasive, real-time evaluation of abdominal organs. However, reference ultrasound data for neotropical marsupials is scarce, which hinders diagnostic interpretation of ultrasound images of these species. The present study aimed to describe the morphological and biometric ultrasound characteristics of the abdominal organs of white-eared opossums (*Didelphis albiventris*) treated at a wildlife rehabilitation center in southern Brazil. Sixteen clinically stable opossums, with an average body mass of 1.4 kg, underwent abdominal ultrasonography using a multifrequency linear transducer (7–12 MHz). We analyzed parameters such as shape, contour, echogenicity, echotexture, and anatomical relationships between adjacent structures, in addition to taking biometric measurements of different abdominal organs. Most structures presented characteristics similar to those described in small domestic dogs and cats. However, species-specific particularities were observed, including decreased renal corticomedullary distinction, slightly coarser renal and prostatic echotexture, and a smaller abdominal aorta diameter. It is concluded that abdominal ultrasonography is an effective method for the anatomical evaluation of white-eared opossums.

## Simple Summary

The white-eared opossum (*Didelphis albiventris*) is a common wild marsupial in South America and is frequently treated in wildlife rehabilitation centers. Despite its importance in veterinary medicine, little is known about the usual arrangement of abdominal organs on ultrasound examination, which hinders the clinical evaluation and diagnosis of diseases in this species. This study describes the ultrasound characteristics of the abdominal organs of clinically healthy white-eared opossums treated at a rehabilitation center in southern Brazil. Sixteen animals underwent abdominal ultrasounds, which allowed for the evaluation of organs such as the urinary bladder, kidneys, spleen, stomach, intestines, liver, adrenal glands, testicles, and prostate. Most structures exhibited ultrasound patterns similar to those observed in domestic dogs and cats, although some species-specific differences were identified. Our results provide important reference information for veterinarians working with wild animals, and may help with the diagnosis, clinical management, and rehabilitation of white-eared opossums.

## 1. Introduction

The increasing occurrence of clinical care for wild animals in triage and rehabilitation centers has increased the demand for effective diagnostic methods applicable to different species [1,2]. Among these species, the white-eared opossum (*Didelphis albiventris*) stands out for its wide distribution in South America and its ecological plasticity and synanthropic behavior. This proximity to anthropized areas increases the frequency of interactions with humans, consequently increasing the need for clinical and emergency care in specialized centers [3]. However, the clinical evaluation of these animals presents significant challenges, especially in identifying internal alterations that are not evident during physical examinations. Thus, diagnostic imaging methods are indispensable in veterinary practice [4].

Ultrasonography is a noninvasive, safe method that can provide real-time assessments of internal organs and is widely used in veterinary medicine for diagnostic purposes [5,6]. Despite its clinical relevance, there are still few reference ultrasonographic descriptions for neotropical marsupials, especially white-eared opossums. Previous studies of this species have focused mainly on anatomical, histological, and radiographic aspects, while abdominal ultrasonographic characterization remains limited and poorly standardized [7,8]. Furthermore, knowledge of normal ultrasonographic patterns is fundamental for the proper interpretation of examinations and for the early identification of pathological changes [9].

Thus, the present study aimed to characterize morphology and establish the ultrasonographic biometric parameters of the abdominal organs of white-eared opossums treated at the Wildlife Triage and Rehabilitation Center of the State University of the Midwest (Cetras-Unicentro). This contributes to the exploratory study of the ultrasonographic findings and improves the clinical and diagnostic evaluation of the species.

## 2. Materials and Methods

This observational, descriptive, and prospective study was approved by the Ethics Committee on Animal Use (CEUA) of the State University of the Midwest (Unicentro, Guarapuava, Brazil), under protocol no. 12/2024, approved on 11 June 2024. The study was conducted from 6 August 2024 to 11 May 2026, using 16 white-eared opossums from urban rescues that were sent to the Wildlife Triage and Rehabilitation Center (Cetras) at the Cedeteg campus of Unicentro in Guarapuava, state of Paraná, Brazil.

The evaluated population consisted of seven males and nine females, classified as six adults and ten juveniles, with an average body mass of 1.4 kg (minimum: 1.0 kg; maximum: 2.3 kg). Age classification was established based on body mass, head circumference, and tail length [10]. Animals were considered clinically stable if they passed a complete physical examination, behavioral assessment, body condition scoring, and complementary laboratory testing, including complete blood counts and fecal parasitological examinations. Only individuals with a body mass greater than 1.0 kg that were suitable for safe physical restraint were included.

Ultrasound examinations were performed on animals in dorsal recumbency using physical restraint by assistants who were previously trained in the handling of wild animals. For one individual, the examination was performed during an anesthetic procedure that had been instituted for a surgical intervention unrelated to the present study. Evaluations took place in a quiet environment with reduced lighting to minimize stress on the animals. For more agitated individuals, the eyes were covered to reduce visual stimuli. The average examination time was approximately 15 min, and all individuals were monitored throughout the procedure.

No water or food fasting was performed before the examinations. However, the evaluations took place at the end of the afternoon period, considering the animals’ feeding routine. Abdominal trichotomy was not routinely performed due to the possibility of returning most individuals to their natural environment. The abdominal region was moistened with 70% alcohol, followed by the application of conductive gel to optimize ultrasound image quality.

Ultrasound examinations were performed at the Veterinary Diagnostic Imaging Sector of the Veterinary Teaching Clinic of the State University of the Midwest (Unicentro), using an Esaote^®^ MyLab 40 Vet device (Esaote, Rio de Janeiro, Brazil) equipped with a multifrequency linear transducer (7–12 MHz). The 12 MHz frequency was used most frequently to provide better resolution in these small animals. Image acquisition was performed by a single experienced veterinarian specializing in veterinary diagnostic imaging.

During the ultrasound evaluations, the veterinarian analyzed the morphological characteristics of the abdominal organs, including their shape, contour, echogenicity, echotexture, and the anatomical relationships between adjacent structures. Biometric measurements were taken of the urinary bladder; the measurements of the urinary bladder wall thickness were taken in a representative region of the wall—extending from the mucosa to the serosa—while maintaining the ultrasound beam perpendicular to the bladder wall in both longitudinal and transverse planes. Urinary bladder volume was measured using the automated Bi-Volume function of the ultrasound system (RES 1.01/ODS 13.10/VET 13.20, MyLab, Esaote^®^), which applies the prolate ellipsoid formula—an established method for estimating the volume of ovoid anatomical structures based on three orthogonal diameters:

V = (π/6) × D1 × D2 × D3

D1 = longitudinal diameter (length); D2 = laterolateral diameter (width); D3 = dorsoventral diameter (height/depth). These three diameters were obtained directly from B-mode ultrasound images; D1 was measured in the longitudinal (sagittal) plane, while D2 and D3 were measured in the transverse plane of the urinary bladder. After the examiner manually positioned the electronic calipers for the three measurements, the device automatically calculated the volume.

Kidneys: The renal length was obtained by identifying the greatest linear distance between the cranial and caudal poles in the longitudinal plane. Spleen: The spleen thickness was measured in the transverse plane at the level of the splenic hilum. Adrenal glands: The measurements included the length (from the cranial pole to the caudal pole) and thickness of the cranial and caudal extremities, obtained in longitudinal and transverse planes.

Stomach: The gastric wall thickness was measured from the luminal mucosal margin to the serosal surface in the longitudinal and transverse planes. Gallbladder: The wall thickness was measured as a thin, echogenic, delimiting line in a representative region on the longitudinal section. The same equation and methodology described for the urinary bladder were used to estimate the volume of the gallbladder. For the duodenum, the wall thickness was measured in the longitudinal plane, from the inner margin of the mucosa to the outer margin of the serosa. Colon: The wall thickness was measured in cross-section, from the inner margin of the mucosa to the outer margin of the serosa. Testicles: The measurements of the craniocaudal and laterolateral axes were obtained in a longitudinal section. Prostate: The measurements of the craniocaudal and laterolateral axes were obtained in a longitudinal section.

The abdominal aortic diameter was measured in the longitudinal plane, caudal to the origin of the left renal artery—taking the maximum intraluminal diameter—for the kidney-to-aorta (R/Ao) ratio. The adrenal glands were evaluated whenever they could be identified by ultrasound. Each measurement obtained in duplicate allows for greater standardization and data reliability.

The ultrasound images were stored digitally on the equipment itself and then transferred to the institutional cloud storage system (PACS-RPACS). This ensured traceability, secure archiving, and the possibility of later reassessment. The data were organized and tabulated in Microsoft Excel^®^ spreadsheets. Descriptive statistical analysis was then performed, including calculating the mean, standard deviation, minimum, and maximum values of the evaluated biometric variables. No inferential analyses were conducted.

## 3. Results

### 3.1. Urinary System

The biometric results for the urinary organs are presented in Table 1. The urinary bladder (n = 14) was identified in the caudoventral region of the abdomen in 87.5% of individuals. In two individuals, visualization was not possible due to fecal retention in the colon, which was located dorsally to the urinary bladder. These two animals were not included in the biometric analysis.

In 87.5% of cases, the urinary bladder had a hyperechoic wall and anechoic, homogeneous intraluminal content with moderate repletion. In two individuals, low bladder distension associated with increased wall thickness was observed. The average bladder wall thickness was 0.12 ± 0.04 cm, with an average volume of 11.30 ± 8.60 mL.

**Figure 1:**
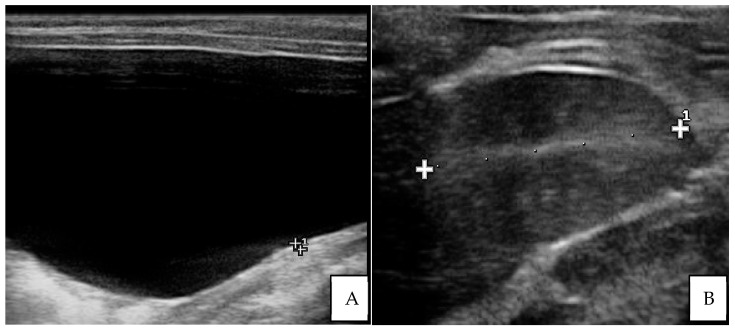
Ultrasound images of the urinary bladder (**A**) and kidney (**B**) in a white-eared opossum (*Didelphis albiventris*) obtained in the longitudinal and transverse planes. In (**A**), the urinary bladder is observed, with a hyperechoic wall delimiting the anechoic intraluminal content corresponding to urine. In (**B**), the kidney is observed, delimiting the renal length. Image (**A**): 1 = urinary bladder wall (thickness). Image (**B**): 1 = kidney (length).

The kidneys (n = 16) were oval and elongated in all individuals evaluated. Topographically, the left kidney was adjacent to the body of the spleen, and the right kidney was near the caudate lobe of the liver (Figure 1).

The renal cortex exhibited greater echogenicity than the hepatic and splenic parenchyma in all individuals evaluated and was classified as hyperechoic. Corticomedullary distinction was observed in 33.33% of individuals. Regarding renal echotexture, all animals presented a slightly coarse pattern.

The average length of the left kidney was 2.50 ± 0.29 cm. The right kidney had an average length of 2.40 ± 0.15 cm.

### 3.2. Digestive System

The biometric results for the organs of the digestive tract are presented in Table 2. The stomach (n = 16) was predominantly located in the xiphoid region of the cranial abdominal region, while the intestinal loops extended through the inguinal and pubic regions.

**Figure 2:**
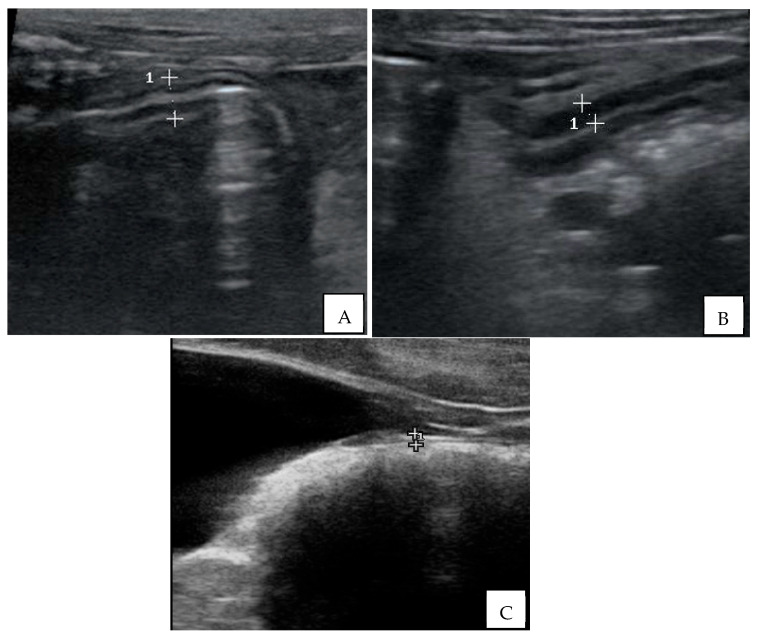
Ultrasound images of the stomach (**A**), duodenum (**B**) and colon (**C**) in a white-eared opossum (*Didelphis albiventris*) obtained in the longitudinal and transverse planes. In (**A**), the stomach is observed, with identification of the gastric wall. In (**B**), the duodenum is observed, showing the thickness of the intestinal wall. In (**C**), the colon is observed, showing the thickness of the intestinal wall. Image (**A**): 1—stomach wall (thickness). Image (**B**): 1—duodenum (thickness). Image (**C**): 1—colon (thickness).

The four layers of the gastric wall were visualized in 81.25% of the evaluated individuals (Figure 2A). Thickening of the gastric submucosa was observed in 13.33% of the cases. In 18.75% of the individuals, the stomach was collapsed or empty, which made delineating the fundus and pylorus difficult.

The intraluminal gastric content was heterogeneous and associated with slight posterior acoustic shadowing and a reverberation artifact. The gastric mucosa and muscular layers were hypoechoic, while the submucosa and serosa were hyperechoic. The average thickness of the gastric wall was 0.21 ± 0.09 cm.

The duodenum (n = 16) had a straight, superficial course along the right lateral abdominal wall. All individuals evaluated had four intestinal layers, including serosa, muscularis, submucosa, and mucosa (Figure 2B). The intestinal lumen had heterogeneous, hyperechoic content. The average duodenal wall thickness in the longitudinal section was 0.20 ± 0.06 cm. The average thickness of the colon wall was 0.22 ± 0.10 cm (Figure 2C).

**Figure 3:**
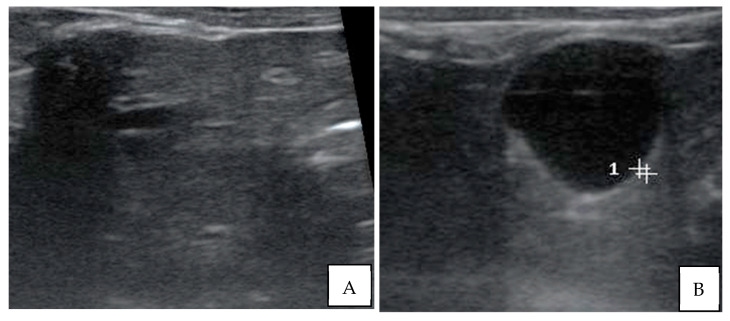
Ultrasound images of the liver (**A**) and gallbladder (**B**) in a white-eared opossum (*Didelphis albiventris*) obtained in the longitudinal and transverse planes. In (**A**), the left portion of the left lateral and medial lobes is observed. In (**B**), the gallbladder is observed, highlighting the wall. Image (**B**): 1—gallbladder wall (thickness).

The liver (n = 16) was identified cranially to the stomach in all individuals evaluated. The left portion of the left lateral and medial lobes was more pronounced, extending to the costal arch (Figure 3A). The hepatic parenchyma was uniformly hypoechoic in 81.25% of individuals compared to the spleen.

The gallbladder (n = 16) was located to the left of the midline in all individuals evaluated. It had a hyperechoic wall and anechoic intraluminal content corresponding to bile (Figure 3B). The average thickness of the gallbladder wall was 0.11 ± 0.03 cm, with an average volume of 0.72 ± 0.41 mL.

### 3.3. Spleen, Adrenal Glands, and Aorta

The biometric results for the spleen, adrenal glands, and abdominal aorta are listed in Table 3.

**Figure 4:**
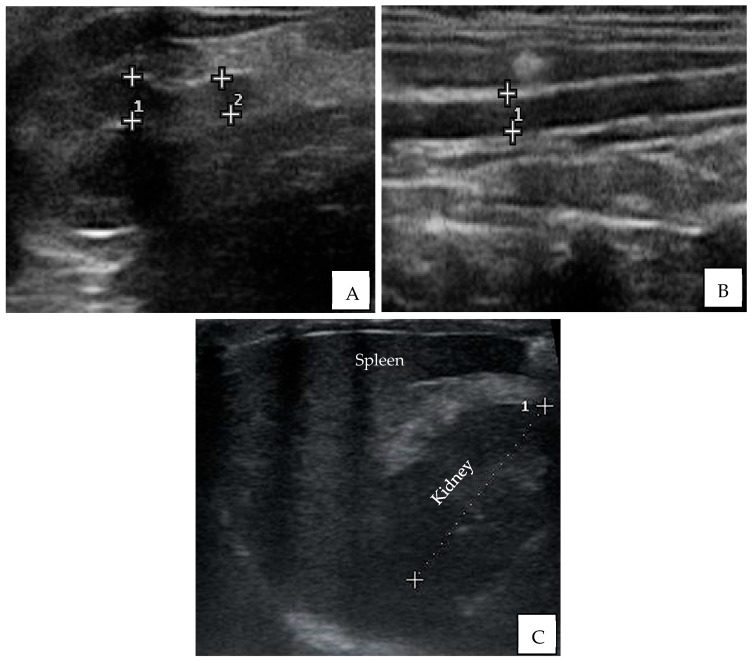
Ultrasound images of the adrenal gland (**A**) and the abdominal aorta (**B**). Spleen adjacent to the left kidney (**C**) in a white-eared opossum (*Didelphis albiventris*) obtained in the longitudinal and transverse planes. In (**A**), the adrenal gland is observed, with distinction between its cranial and caudal ends. In (**B**), the abdominal aorta is observed, showing its hyperechoic wall. In (**C**), the location of the spleen in relation to the left kidney is shown. Image (**A**): adrenal gland 1 = caudal end and 2 = cranial end (thickness). Image (**B**): 1 = abdominal aorta (diameter). Image (**C**): 1 = kidney (length).

The spleen (n = 11) was located caudodorsally relative to the left kidney (Figure 4). It was triangular and elongated, with a smooth, regular, echogenic capsule and hyperechoic parenchyma relative to the liver, as well as a homogeneous echotexture. The average splenic thickness in the transverse plane was 0.73 ± 0.20 cm.

The left adrenal gland (n = 6) was identified in 37.5% of individuals, while the right adrenal gland (n = 3) was visualized in 18.75% of cases. The average thickness of the cranial end of the left adrenal gland was 0.36 ± 0.09 cm. The cranial end of the right adrenal gland had an average thickness of 0.39 ± 0.06 cm.

The caudal end of the left adrenal gland had an average thickness of 0.26 ± 0.06 cm, and the right adrenal gland had an average thickness of 0.28 ± 0.04 cm. The average left length of the adrenal glands was 1.23 ± 0.38 cm, and the average right length of the adrenal glands was 1.19 ± 0.26 cm.

The adrenal glands were located in the retroperitoneal space, medially to the kidneys. The left adrenal gland was located adjacent to the lateral face of the abdominal aorta, and the right adrenal gland was located near the caudal vena cava. Both presented an ovoid, cylindrical shape and hypoechoic parenchyma (Figure 4A).

The abdominal aorta (n = 16) was identified in the dorsal longitudinal plane of the abdomen, medially to the left kidney. It had a tubular appearance with anechoic content, hyperechoic walls, and a pulsatile characteristic with responsiveness to color Doppler (Figure 4B). The observed diameter was 0.35 ± 0.06 cm.

### 3.4. Male Reproductive Organs

The biometric results of the male reproductive organs are presented in Table 4. Of the 16 individuals evaluated, seven were males.

**Figure 5:**
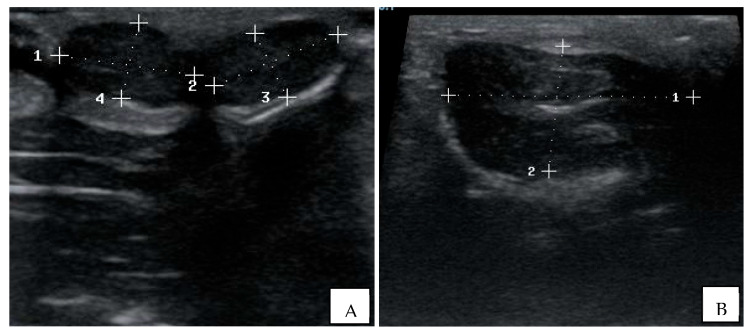
Ultrasound images of the testes (**A**) and prostate (**B**) in a white-eared opossum (*Didelphis albiventris*). In (**A**), the right and left testicles are observed, showing the testicular midline. In (**B**), the prostate is observed with a bilobed aspect. Image (**A**): testes 1–2= laterolateral axis; 3–4 = craniocaudal axis. Image (**B**): prostate 1 = laterolateral axis; 2 = craniocaudal axis.

The testicles (n = 7) were identified inside the scrotum, presenting an oval morphology, intermediate echogenicity, and a slightly coarse echotexture (Figure 5A). The testicular mediastinal line, characterized by a hyperechoic linear structure, was visualized in 57.14% of the individuals evaluated.

The right testicle (adult) had an average dimension of 1.82 ± 0.26 cm in the craniocaudal axis and 0.95 ± 0.04 cm in the laterolateral axis. The left testicle (adult) had an average of 1.85 ± 0.21 cm in the craniocaudal axis and 0.90 ± 0.04 cm in the laterolateral axis.

The prostate (n = 4) was identified in the transabdominal caudal region, located in the pelvic canal. A bilobed conformation was observed in 100% of the adult individuals evaluated. The prostate presented a predominantly coarse echotexture, echogenicity similar to splenic parenchyma, and regular, well-defined margins (Figure 5B).

Regarding the ultrasonographic findings of the male reproductive organs, which were analyzed according to sexual maturity stage, differences were observed between adult (n = 3) and juvenile (n = 4) males. The prostate was visualized in all adults (3/3), but in only one of the juveniles (1/4), suggesting less prostatic development and smaller size in immature animals, which hinders their identification via ultrasound. In relation to testicular echotexture, a coarse appearance was observed in most adults and in some young animals, whereas the remaining young animals exhibited a fine, homogeneous echotexture, a pattern more consistent with that described for domestic dogs and cats. Such variations reinforce the influence of the developmental stage on the ultrasonographic characteristics of the reproductive organs.

However, given the small number of individuals per group and the fact that age classification was based on body mass and external morphology, rather than direct assessment of gonadal maturity, the results should be interpreted descriptively and viewed with caution.

## 4. Discussion

### 4.1. Urinary System

The urinary bladder exhibited ultrasonographic characteristics similar to those reported for domestic mammals, especially cats, including a hyperechoic wall and homogeneous, anechoic intraluminal content. In individuals with low bladder filling, an increase in wall thickness was observed, a finding compatible with the description by Sutherland-Smith [11], who reports a direct influence of bladder distension on urinary wall thickness.

The inability to visualize the urinary bladder in two individuals may be related to fecal retention in the colon, a structure located dorsally to the urinary bladder, which makes ultrasonographic delimitation difficult, as described by Muller et al. [12].

The kidneys had an oval, elongated morphology similar to that observed in opossum pups by Cavalcanti et al. [4] and to anatomical patterns described for domestic mammals by D’Anjou [13]. The observed renal topography was also similar to the anatomy described in small domestic animals.

The superior renal cortical echogenicity compared to the liver and spleen, observed in all individuals, corroborates the findings of Yeager and Anderson [14], who suggest that cortical lipid vacuoles contribute to greater ultrasound reflection. However, the reduced corticomedullary definition and slightly coarse echotexture observed in most individuals differ from the pattern described for domestic cats and wild felines [12,13].

Although the renal lengths obtained were lower than those reported for domestic cats [15], the average kidney/aorta ratio was 8.14, which remains within the normal parameters for small domestic dogs according to D’Anjou [13]. These results imply that despite the smaller absolute dimensions of the kidneys, their proportions in relation to vascular caliber are compatible with physiological parameters described for small domestic dogs. As a function of the sample size, the presented values are preliminary descriptive data.

### 4.2. Digestive System

The anatomical location of the stomach and intestinal loops observed in the present study was similar to the anatomical description of small domestic dogs [16]. Visualization of the four gastric layers in most individuals indicates adequate ultrasound definition of digestive structures. However, the presence of a collapsed stomach made anatomical delimitation difficult in some animals, as previously described by Penninck [17].

The ultrasonographic pattern of the gastric layers exhibited a similar echogenicity distribution to that observed in domestic cats: hypoechoic mucosa and muscularis, and hyperechoic submucosa and serosa. The average thickness of the gastric wall was also compatible with the parameters reported for domestic cats [17].

The descending duodenum exhibited sufficient ultrasonographic definition in all evaluated individuals, enabling full identification of the intestinal layers. The biometric values obtained for duodenal thickness were compatible with the ranges described for domestic mammals, particularly the lower limits reported for cats [17].

The average thickness of the colon wall was slightly higher than the values usually described for domestic animals. However, the observed range of variation partially overlapped with the parameters described for cats [17,18].

The observed hepatic topography was similar to that described in small dog breeds and cats, particularly with regard to the predominance of the left portion of hepatic lobes and extension to the costal arch [13,19]. The hepatic parenchyma was predominantly hypoechoic and homogeneous in most individuals evaluated.

The gallbladder exhibited ultrasonographic characteristics consistent with those described for domestic mammals, including a hyperechoic wall and anechoic luminal content corresponding to bile [13,16]. The biometric values obtained for gallbladder wall thickness showed low variability among the evaluated individuals, suggesting a relatively uniform pattern for the species.

### 4.3. Spleen, Adrenal Glands, and Abdominal Aorta

The spleen had an anatomical location, shape, and ultrasonographic characteristics similar to those of domestic mammals. These characteristics included a regular echogenic capsule and homogeneous echotexture. However, the observed splenic thickness was lower than that described for *Leopardus guttulus* by Muller et al. [12], though it remained within 3 the reference ranges reported for domestic cats by D’Anjou [13].

The abdominal aorta presented an ultrasonographic pattern consistent with the arterial vessels of domestic mammals, including a tubular aspect, anechoic content, and responsiveness to color Doppler. However, the observed biometric values were lower than the minimum limits described for domestic dogs by Meirelles and Aguilera [20], suggesting a possible anatomical peculiarity related to the body size and vascular physiology of the species.

The ultrasonographic identification of the adrenal glands was limited, particularly the right adrenal gland; this was possibly due to the small anatomical size of the structures and their proximity to adjacent organs. This is a factor frequently described in ultrasonographic examinations of small mammals.

The visualized adrenal glands presented a morphology similar to that described by Dyce et al. [16], characterized by an elongated, asymmetrical, and irregular shape. The observed topography also agreed with previously established anatomical descriptions, including a retroperitoneal location medial to the kidneys, a left adrenal gland adjacent to the abdominal aorta, and a right adrenal gland close to the caudal vena cava.

The biometric values (thickness) of the adrenal glands were compatible with the reference ranges described for small domestic dogs by Meirelles and Aguilera [20]. Furthermore, the hypoechoic pattern of the adrenal parenchyma corroborates observations by Graham [21] in domestic mammals. The small sample size was a limitation of this study; therefore, the presented values are preliminary descriptive data.

### 4.4. Male Reproductive Organs

The testicles presented a similar anatomical location and shape to those described for domestic dogs. They were identified inside the scrotum and had an oval morphology. However, the slightly coarse echotexture observed in the evaluated individuals differs from the homogeneous pattern described by Hecht [22] for domestic cats.

Visualization of the mediastinum testis in some individuals demonstrates similarity to the ultrasonographic pattern described for dogs, in which this structure is characterized as a central hyperechoic line. The absence of this structure in some individuals may be related to the reduced size of the testicles, their anatomical positioning, or technical limitations inherent in ultrasonographic examinations of wild animals.

The observed testicular dimensions were compatible with those described by Feliciano, Oliveira and Vicente [23] and Hecht [22] for small domestic dogs, suggesting relative biometric proportionality between *Didelphis albiventris* and small domestic mammals.

The prostate presented a caudal transabdominal location similar to that described for domestic dogs. The bilobed aspect observed in some individuals also agrees with the anatomical pattern described for dogs. However, the predominantly coarse echotexture observed in the present study differs from the homogeneous description reported by Feliciano, Oliveira and Vicente [23] and Hecht [22]. However, given the small number of individuals per group and the fact that age classification was based on body mass and external morphology, rather than direct assessment of gonadal maturity, the results should be interpreted descriptively and viewed with caution.

In general, the prostatic ultrasonographic findings showed partial similarity to those described for small dogs with body mass of less than 10 kg, especially in relation to the dimensions and morphological structure of the gland [23]. The small sample size was a limitation of this study; therefore, the presented values are preliminary descriptive data.

## 5. Conclusions

This study demonstrated that ultrasonography is an effective tool for the anatomical and biometric assessment of the major abdominal organs of the white-eared opossum. It was possible to identify and characterize the structures evaluated, establishing parameters regarding organ echotexture, location, and dimensions. These results represent a significant scientific contribution regarding the species, and may aid future research.

The ultrasonographic findings of this study revealed that the white-eared opossum (*Didelphis albiventris*) exhibits anatomical and ultrasonographic characteristics that are partially similar to those of small breeds of domestic dogs, cats, and wild felines, though important differences were found. The kidneys were smaller than those of small domestic animals, but the kidney/aorta ratio remained within normal parameters for dogs. Additionally, the renal cortex appeared more hyperechoic and exhibited a coarser echotexture. The spleen thickness was similar to that observed in dogs and cats, although it showed slightly lower echogenicity. In the male reproductive system, the prostate had a similar appearance to that described in domestic dogs, but with a coarser echotexture.

The small number of animals is a limitation of the study; however, these results provide important information for veterinarians working with wild animals, and may help with the diagnosis, clinical management, and rehabilitation of white-eared opossums (*Didelphis albiventris*).

## Figures and Tables

**Table 1:** Biometric measurements of the urinary tract of white-eared opossum (*Didelphis albiventris*).

Organ	Mean ± SD	Minimum	Maximum
Urinary bladder wall * (thickness; cm)	0.12 ± 0.04	0.10	0.21
Urinary bladder volume * (mL)	11.30 ± 8.60	3.30	23.31
Left kidney ** (length; cm)	2.50 ± 0.29	1.85	3.45
Right kidney ** (length; cm)	2.40 ± 0.15	1.76	3.33

* n = 14 animals; ** n = 16 animals.

**Table 2:** Biometric measurements of the digestive system of white-eared opossum (*Didelphis albiventris*).

Organ	Mean ± SD	Minimum	Maximum
Stomach wall (thickness; cm) *	0.21 ± 0.09	0.14	0.43
Duodenum (thickness; cm) *	0.20 ± 0.06	0.12	0.30
Colon (thickness; cm) *	0.22 ± 0.10	0.12	0.37
Gallbladder wall (thickness; cm) *	0.11 ± 0.03	0.08	0.17
Gallbladder volume (mL) *	0.72 ± 0.41	0.33	1.20

* n = 16 animals.

**Table 3:** Biometric measurements of the spleen, abdominal aorta, and adrenal glands of white-eared opossum (*Didelphis albiventris*).

Organ	Mean ± SD	Minimum	Maximum
Spleen (thickness; cm) *	0.73 ± 0.20	0.51	1.52
Left adrenal gland—cranial end (thickness; cm) **	0.36 ± 0.09	0.12	0.47
Right adrenal gland—cranial end (thickness; cm) ***	0.39 ± 0.06	0.32	0.44
Left adrenal gland—caudal end (thickness; cm) **	0.26 ± 0.06	0.19	0.29
Right adrenal gland—caudal end (thickness; cm) ***	0.28 ± 0.04	0.25	0.31
Left adrenal gland (length; cm) **	1.23 ± 0.38	0.80	1.50
Right adrenal gland (length; cm) ***	1.19 ± 0.26	0.66	1.48
Abdominal aorta (diameter; cm) ****	0.35 ± 0.06	0.31	0.40

* n = 11 animals; ** n = 6 animals; *** n = 3 animals; **** n = 16 animals.

**Table 4:** Biometric measurements of the male reproductive organs of white-eared opossum (*Didelphis albiventris*).

Organ	Mean ± SD (cm)	Minimum (cm)	Maximum (cm)
Right testicle (adult; craniocaudal axis × laterolateral axis) *	1.82 ± 0.26 × 0.95 ± 0.04	1.57 × 0.91	2.08 × 0.99
Left testicle (adult; craniocaudal axis × laterolateral axis) *	1.85 ± 0.21 × 0.90 ± 0.04	1.62 × 0.87	2.06 × 0.95
Right testicle (juvenile; craniocaudal axis × laterolateral axis) **	1.37 ± 0.22 × 0.75 ± 0.13	1.27 × 0.55	1.51 × 0.83
Left testicle (juvenile; craniocaudal axis × laterolateral axis) **	1.34 ± 0.15 × 0.67 ± 0.11	1.22 × 0.50	1.49 × 0.75
Prostate (adult; craniocaudal axis × laterolateral axis) *	1.97 ± 0.59 × 1.37 ± 0.21	1.55 × 0.95	2.84 × 1.51

* n = 3 animals; ** n = 4 animals.

## Data Availability

The data presented in this study are available from the corresponding author upon reasonable request.
